# Immunogenicity, security and protection against small ruminant lentivirus (SRLV) challenge in sheep, induced by intranasal immunization with a recombinant Sendai virus vector expressing SRLV gag-P25

**DOI:** 10.1080/01652176.2025.2556492

**Published:** 2025-09-17

**Authors:** Álex Gómez, Idoia Glaria, Irati Moncayola, Irache Echeverría, Ana Rodríguez-Largo, Ignacio de Blas, Estela Pérez, Marta Pérez, Sergio Villanueva-Saz, Benhur Lee, Alicia de Diego, Ricardo de Miguel, Lluís Luján, Ramsés Reina

**Affiliations:** a Departamento de Patología Animal, Universidad de Zaragoza, Zaragoza, Spain; b Instituto Agroalimentario de Aragón-IA2, Universidad de Zaragoza, Zaragoza, Spain; c Instituto de Agrobiotecnología (CSIC-Gobierno de Navarra), Mutilva Baja, Spain; d Departamento de Agronomía, Biotecnología y Alimentación, Universidad Pública de Navarra, Pamplona, Spain; e Servei de Diagnòstic de Patologia Veterinària, Departament de Sanitat i Anatomia Animals, Universitat Autònoma de Barcelona, Barcelona; f Departamento de Anatomía, Embriología y Genética Animal, Universidad de Zaragoza, Zaragoza, Spain; g Department of Microbiology, Icahn School of Medicine at Mount Sinai, New York, NY, USA; h Centro de Investigación Biomédica de Aragón (CIBA), Instituto Aragonés de Ciencias de la Salud (IACS), Zaragoza, Spain; i AnaPath Services GmbH, Liestal, Switzerland

**Keywords:** Sendai, viral vector, vaccine, intranasal, small ruminant lentiviruses

## Abstract

Small ruminant lentiviruses (SRLV) are responsible for significant economic losses in sheep and goat farming; however, effective vaccination strategies remain unavailable. This study evaluated the immunogenicity, safety, and protective efficacy of a recombinant Sendai virus vector (SeV) expressing SRLV *gag*-P25 (rSeV-GFP-P25) in lambs. Twenty-one SRLV-negative lambs were divided into three groups and inoculated intranasally thrice with culture medium (group 1); SeV-GFP (group 2) or rSeV-GFP-P25 (group 3). Lambs were challenged with homologous SRLV at 16 weeks post-first immunization. Clinical and hematological parameters, antibody responses, SRLV viral loads in peripheral blood mononuclear cells (PBMCs) and target tissues, histopathological and histomorphometric analyses, assisted with artificial intelligence, of interstitial pneumonia were assessed. No clinicopathological alterations were observed, except for a transient temperature increase in group 3 post-first immunization. Group 2 showed mild SeV-neutralizing antibodies, while rSeV-GFP-P25 (group 3) induced negligible SRLV-specific antibody responses. Group 3 exhibited higher SRLV DNA copies in PBMCs but lower in most SRLV target tissues compared to control groups, with no SRLV DNA detected in spleen and bone marrow. Histomorphometry revealed reduced alveolar septal thickening in group 3, indicating partial protection against early SRLV-associated interstitial pneumonia. These results warrant further investigation into cellular immunity and long-term protection.

## Introduction

Small ruminant lentiviruses (SRLV) are responsible for a significant global infection in sheep and goats, leading to decreased milk and meat production and silently impacting the economy of farms (Juste et al. 2020; de Miguel et al. 2021). The main lesions caused by SRLV include slow and progressive pneumonia, mastitis, arthritis and/or encephalitis (Sigurdsson 1954; Crawford et al. 1980; Minguijón et al. 2015). SRLV ability to evade the immune system through mechanisms such as integration into the host cell genome as a provirus, genetic viral diversity, variability in cytotoxic T lymphocytes (CTLs)-epitopes, presence of low levels of cell-free viral particles in the blood, among others allow the development of the disease (Haase and Varmus 1973; Lichtensteiger et al. 1993; Reina et al. 2006, 2009; Haflidadóttir et al. 2008; Blacklaws 2012; Ramírez et al. 2013; Castañeda-Montes et al. 2023). As a result, SRLV cause persistent infections that can increase susceptibility to secondary infections, finally affecting animal production (Junkuszew et al. 2016; Gomez-Lucia et al. 2018; Urbańska et al. 2022).

The main risk factors for horizontal transmission rely on prolonged housing periods, intensive management systems, and animal crowding (Leginagoikoa et al. 2009; Illius et al. 2020). During the last decades, the increasing number of intensively managed flocks and the lack of treatments or vaccines have led to a rise in SRLV seroprevalence sometimes reaching 90–100% (Kaba et al. 2013; Olech et al. 2019; de Miguel et al. 2021; Jacob-Ferreira et al. 2023). Current strategies for controlling SRLV horizontal transmission involve early serological identification and segregation of infected animals, whereas pasteurization prevents SRLV lactogenic transmission (Peterhans et al. 2004; Gjerset et al. 2009; Herrmann-Hoesing 2010; Jacob-Ferreira et al. 2023). However, successful control programs are rare, and no SRLV eradication scheme has been documented until date, with the exception of Icelandic stamping out policies (Peterhans et al. 2004;), further highlighting the need for developing vaccination strategies.

Multiple vaccination strategies against SRLV have been explored, including live attenuated, inactivated, subunit, plasmid DNA and *Vaccinia* virus-based vaccines. However, the genetic variability of SRLV and the arrangement of immune evasion mechanisms pose challenges in developing effective vaccines (Reina et al. 2013). Attenuated vaccines may revert to virulence since recombination events are common and inactivated vaccines can increase lesion development (Nathanson et al. 1981; Cutlip et al. 1987; Harmache et al. 1996, 1998; Kemp et al. 2000; Nenci et al. 2007). Although subunit vaccines can induce specific neutralizing antibodies, some surface glycoproteins favored SRLV replication (Kemp et al. 2000; Nenci et al. 2007). Plasmid DNA immunizations encoding *gag* or *env* genes delivered by gene gun and *Vaccinia*-based vectors encoding *env* gene have shown a strong humoral response, earlier restriction of the virus replication and the improvement of clinical signs (Cheevers et al. 2000; Beyer et al., 2001; González et al. 2005). Moreover, heterologous nasal and systemic prime-boost regimes by plasmid DNA and *Vaccinia*-based vectors, both encoding *gag* and *env* genes, have demonstrated a reduction of proviral load and lesion severity (Niesalla et al. 2009; Reina et al. 2009). However, these vaccines have shown highly variable degrees of protection against SRLV infection and require specific devices for inoculation, resulting in an imbalance in terms of cost-benefit.

Sendai virus (SeV)-based vectors have obtained promising safety and efficacy results in the development of vaccine prototypes for lentiviruses, such as human immunodeficiency virus and simian immunodeficiency virus (Kano et al. 2000; 2002; Gómez and Reina 2025b). In sheep, SeV has shown efficient transgene expression, robust type I IFN-mediated innate immune response activation and partial protection against SRLV infection in sheep cells *in vitro* (Griesenbach et al. 2011; De Pablo-Maiso et al. 2020; Gómez et al. 2025a). Among SRLV-encoded proteins, *gag*-derived proteins are those more conserved, with the P25 protein being a major core protein and the preferred choice to develop indirect ELISA tests to detect antibodies in infected animals (Echeverría et al., 2020). SRLV P25 protein has shown immunogenic properties, able to generate high levels of functional antibodies (Narayan and Clements 1989; Reyburn et al. 1992; Castañeda-Montes et al. 2023). Our group previously generated a recombinant SeV vector expressing SRLV P25 protein (rSeV-GFP-P25) that has shown a robust innate immune response activation and high restriction of SRLV infection in sheep cells *in vitro*. This recombinant SeV has induced an efficient transgene expression (P25) in epithelial and dendritic cells of nasal mucosa after 48 h of intranasal inoculation in sheep, suggesting potential as a vaccine candidate against SRLV infection (Gómez et al. 2025a).

This study aimed to test the immunogenicity, security and protection induced by rSeV-GFP-P25 in lambs, after challenge with homologous SRLV strain.

## Material and methods

### Cells and viruses

Ovine skin fibroblasts (OSF) and human embryonic kidney cells (HEK293T) were incubated at 37 °C with 5% CO_2_ in Dulbecco’s modified Eagle’s medium (DMEM) (Deltalab, Spain) supplemented with 10% heat-inactivated fetal bovine serum (FBS), 1% L-glutamine and 1% antibiotic/antimycotic mix (Sigma Aldrich, St. Louis, Missouri, USA).

Previously-rescued SeV-GFP and rSeV-GFP-P25 were propagated and titrated on HEK293T cells by fluorescence examination (Nikon Eclipse TE300) in 96-well culture plates using the Reed-Muench method (Reed and Muench, 1938; Gómez et al. 2025a). SRLV stocks from genotype A (strain EV1) (Sargan et al. 1991) were propagated and titrated on OSF, as previously described and used for the challenge (Gómez et al. 2025a). The titer of these three viruses (SeV-GFP, rSeV-GFP-P25 and EV1) was calculated as 50% tissue culture infectious dose per milliliter (TCID_50_/ml).

### Animals and experimental groups

Twenty-one 9–10 month old male, *Rasa Aragonesa* breed lambs were selected from a long-term SRLV seronegative flock. Negative SRLV infection status was confirmed in lambs and their ewes using: 1) two commercial ELISAs: Elitest-MVV (Hyphen-Biomed, France) and Eradikit^™^ SRLV Screening kit (IN3 diagnostic, Italy); 2) a nested PCR with primers amplifying *gag* and *pol* genes (Fw 1 Gag: 5′-TGGTGARKCATAGMTAGAGACATGG-3′; Fw 2 Gag: 5′-CAAACWGTRGCAATGCAGCATGG-3′; Rv 1 Pol: 5′-CATAGGRGGHGCGGACGGCASCA-3′; Rv 2 Pol: 5′-GCGGACGGCASCACACG-3′) (Grego et al. 2002) and 3) a previously-described conventional PCR with primers amplifying an inner region of the *gag* gene (Fw: 5′-TGACAGAAGGAAATTGTYTRTGG-3′; Rv: 5′-GGCATCATGGCTAATACTTCTAA-3′) (Rimstad et al. 1993). Lambs were lodged at the experimental facilities for animal research of the University of Zaragoza and homogeneously distributed by weight into three experimental groups (7 animals each), that were kept in isolated pens with separate feeders and drinkers. Lambs were sterilized prior to the onset of the experimental procedures.

### Immunization and challenge

**Figure 1.**
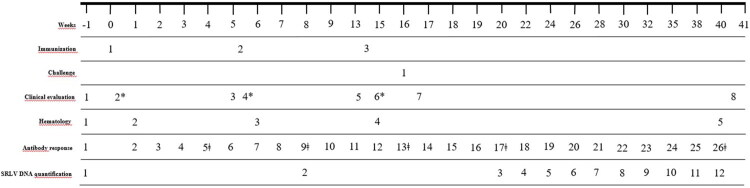
Schedule of immunizations, challenge and methods performed during the experiment. *temperature was measured 24 h pre- and 24, 48 and 72 h after each immunization. Antibody response was measured using Elitest-MVV (Hyphen-Biomed, France). ^ǂ^Seroneutralization assay against SRLV (strain EV1) was performed at 4 weeks post first immunization (wpi), 3 weeks after the second immunization (8 wpi) and 2 weeks after the third immunization (16 wpi) together with 4 and 24 weeks after challenge (20 and 40 wpi). Seroneutralization against SeV-GFP was performed 2 weeks after the third immunization (16 wpi). SRLV DNA quantification was measured using qPCR from isolated peripheral blood mononuclear cells.

Three identical intranasal immunizations at 0, 5 and 14 weeks post-first immunization (wpi) were performed, using a nebulizer (Figure 1). Group 1 (control) was inoculated with 1 ml of DMEM. Groups 2 and 3 were inoculated with 1 ml of 10^3^ TCID_50_/animal of SeV-GFP or rSeV-GFP-P25, respectively. The challenge was performed in all groups at 16 wpi by intra-tracheal inoculation of 10^3^ TCID_50_ SRLV strain EV1 (Sargan et al. 1991) suspended in 1 ml of DMEM, as previously described (Torsteinsdóttir et al. 2003; McNeilly et al. 2007).

### Clinical evaluation

Complete clinical examinations were carried out one week before and after the three immunizations, challenge, and prior to the end of the study (Figure 1). Clinical parameters studied included: body condition, rectal temperature, heart and respiratory rates, inspection of nostrils, palpation of retropharyngeal, submandibular and prescapular lymph nodes, and the evaluation of carpal joints. Specifically, rectal temperature was measured 24 h pre- and 24, 48 and 72 h after each immunization.

### Hematological analysis

Whole blood samples were collected from the jugular vein into 4 ml EDTA anticoagulant tubes at different time points throughout the experiment (Figure 1). Hematology analysis was performed with an IDEXX ProcyteDx automatic hematology counter (IDEXX laboratories, Westbrook, ME, USA). Assessed parameters included erythrocytes (M/μl), hemoglobin (g/dl), hematocrit (%), platelets (K/μl), mean corpuscular volume (MCV; fl), mean corpuscular hemoglobin (MCH; pg), mean corpuscular hemoglobin concentration (MCHC; g/dl), reticulocytes (K/μl), leukocytes (K/ml), neutrophils (K/μl), lymphocytes (K/μl), monocytes (K/μl), basophils (K/μl), and eosinophils (K/μl).

### SeV antibodies

A single seroneutralization assay against SeV-GFP was performed 2 weeks after the third immunization (16 wpi) (Figure 1). HEK293T cells were plated on 96-well plates at a density of 10^4^ cells per well and incubated overnight at 37 °C and 5% CO_2_. Sera were heat-inactivated for 30 min at 56 °C and serially diluted (1:3) in DMEM from 1:3 to 1:6561. SeV-GFP (100 TCID_50_) was added to sera dilutions and incubated overnight at 4 °C. Plated HEK293T cells were washed and incubated with serum-virus dilutions. DMEM or SeV-GFP at 0.1 multiplicity of infection (MOI) were used as negative and positive controls, respectively. Serum-virus dilutions were removed and replaced by DMEM 24 h after incubation at 37 °C and 5% CO_2_. After 7 days of incubation, HEK293T cells were observed by fluorescence microscopy (Nikon Eclipse TE300) and detection of virus-encoded GFP fluorescence was considered as a positive infection. Neutralizing antibodies titer was defined as the Log_10_ of the inverse serum dilution at which 50% of the cultures showed GFP fluorescence (Log_10_ TCID_50_/mL).

### SRLV antibodies

Specific anti-SRLV antibodies were measured at different time points (Figure 1) with Elitest-MVV ELISA (Hyphen-Biomed, France), based on SRLV P25 recombinant protein and a transmembrane synthetic peptide both derived from genotype A (Saman et al. 1999). Cut-off was calculated using the following formula for each ELISA plate: [(positive control OD_450_ – negative control OD_450_)/4] + negative control OD_450_. Animals with optical density ratios (OD_450_) higher than 0.25 were considered positive. For the seroneutralization assay against SRLV (strain EV1), sera obtained after each immunization (4, 8, 16 wpi), after challenge (20 wpi) and prior to euthanasia (40 wpi) were used (Figure 1). Seroneutralization assay was performed as described for SeV-GFP, using 100 TCID_50_ of SRLV strain EV1 in each sera dilution. However, 7 days after incubation, OSF plates were fixed with methanol:acetone (1:1) for 5 min at room temperature, washed and stained with Giemsa (Giemsa Stain, Sigma-Aldrich^®^) for the visualization of syncytia, considered an indication of infection (positive result). Neutralizing antibody titer was calculated as described above.

### SRLV DNA quantification

Peripheral blood mononuclear cells (PBMCs) were isolated from whole blood samples at different time points (Figure 1). Isolation was performed on a Ficoll-Hypaque gradient (δ = 1.077; Lymphoprep Axis-Shield). PBMC pellets were lysed and DNA extraction was performed manually (E.Z.N.A.^®^ Blood DNA Kit, Omega Bio-tek). In addition, SRLV target tissues were collected at necropsy and stored at −80 °C until use. These samples included bronchoalveolar lavage (BAL), lung (all lobes), mediastinal lymph node, spleen, bone marrow, cerebrum, spinal cord and synovial membrane (Olech 2023). Around 20 mg of each sample was homogenized with steel beads in a vibration grinding mill (Mikro-Dismembrator U, Sartorius AG, Germany) coupled with a magnetic bead-based method for DNA extraction and purification (NucleoMag^®^DNA-MagnetaPure32, Macherey-nagel, Germany). For both, PBMCs and tissues, quantity and purity of the extracted nucleic acids were evaluated with a microvolume ultraviolet-visible spectrophotometer (NanoDrop OneC, Thermo Scientific^®^, Waltham, MA, USA). Proviral load of SRLV (strain EV1) was measured by qPCR using AriaMx Realtime PCR System (Agilent Technologies, USA) applying 250 ng of DNA and specific primers (Fw: 5′-CTCCTTGCAGGCCACAATG −3′; Rv: 5′-GCTGCTTGCACTGTCTCGG-3′) and probe (6-FAM-TGCCTTATGTGTAGTCAGC-TAMRA) (Gómez et al. 2025a). A standard curve (R^2^: 0.98) was used to determine the SRLV DNA copies (Log_10_ SRLV copies).

### Gross and histopathological analysis

Lambs were euthanized 41 weeks after the beginning of the experiment (25 weeks post-challenge) (Figure 1). At necropsy, macroscopic SRLV-associated lung lesions were evaluated and categorically scored as 0 (absence) and 1 (compatible). To evaluate the tissue damage after SRLV challenge, the following tissues were sampled: lung (all lobes), mediastinal lymph node, spleen, bone marrow, cerebrum, cerebellum, brain stem, spinal cord (cervical, thoracic and lumbar) and synovial membrane (Olech 2023). To evaluate the tissue response to SeV-GFP and rSeV-GFP-P25, additional SeV target tissues such as nasal mucosa, trachea, nasopharynx-associated lymphoid tissue (NALT) and retropharyngeal lymph nodes were also collected (Griesenbach et al. 2011; Gómez et al. 2025a). Tissues were fixed in 10% neutral-buffered formalin, routinely processed through graded alcohols and embedded in paraffin wax. Sections of 4 µm thick were stained with hematoxylin and eosin (HE). Microscopic severity of SRLV-associated lung lesions was assessed using a semiquantitative score ranging from 0 to 3 (0: absence of lesions; 1: mild; 2: moderate; 3: severe) (Supplementary Table S1). Microscopic analysis was independently performed by three board-certified (Dipl. ECVP) pathologists.

### Histomorphometry analysis

To evaluate the thickening of alveolar septa, interpreted as interstitial pneumonia, histomorphometry analyses were performed on HE stained slides from all animals. Slides were whole slide imaged scanned (WSI) by an Axio Z1 slides scanner (Zeiss, Germany) using a Colibri 7 camera and the 20x objective. Histomorphometry evaluation was conducted by artificial intelligence (AI) using QuPath, quantitative pathology and bioimage analysis software, version 0.5.1 (Bankhead et al. 2017). Pulmonary parenchyma was annotated as the ROI (Region of Interest) excluding the airways (bronchi/bronchiole) and pulmonary vessels. A machine-learned pixel classifier of random trees was employed for the segmentation of alveolar septa and alveolar lumen and the area occupied by the alveolar septa and alveolar lumen were measured. Results were expressed as percentage of pulmonary parenchyma (alveolar septa).

### Statistical analysis

Data were analyzed using IBM SPSS 26.0 for Windows^®^. Categorical variables (ELISA and qPCR positive/negative results and macroscopic SRLV-associated lung lesions) were described using absolute (*n*) and relative frequencies (%). The association of variables with experimental groups was assessed using the Likelihood Ratio test (LR). Dependence between categories of analyzed variables was confirmed by calculating the adjusted standardized residuals (ASR). Under the assumption that the two categorical variables were independent (the null hypothesis), and using a confidence level of 0.95, an ASR greater than 1.96 signified that the number of cases was significantly higher than expected if the null hypothesis held true. Conversely, an ASR less than 1.96 indicated that the number of cases was significantly lower than expected, meaning the category was underrepresented (Agresti 2006). Histopathology severity, a discrete quantitative variable was described as indicated above and compared with different groups performing Kruskal-Wallis test followed by pairwise comparisons among experimental groups using Dunn post-hoc test. Continuous variables (clinical evaluation, OD_450_, titer of neutralizing antibodies, viral load and % pulmonary parenchyma) were described using mean and standard deviation (SD). After testing the normal distribution of the data by Shapiro–Wilk’s test, for normally distributed variables Levene’s test was applied. For variables with equal variances, one-way analysis of variance (ANOVA) was applied. After ANOVA analysis, Bonferroni correction was applied in multiple pairwise comparisons. For normal variables with unequal variances, Welch’s *t*-test was used and multiple pairwise comparisons were performed using Games Howell post-hoc test. For non-normal distributed variables, Kruskal–Wallis test was conducted and multiple pairwise comparisons were applied by Dunn post-hoc test. To compare the values of the same variant over time (clinical evaluation, OD_450_, titer of neutralizing antibodies and viral load) paired Sample *T*-Test (normally distributed variables) or Wilcoxon test (non-normal variables) were used. Statistical significance was set at *p* < 0.05 and represented as *p < 0.050, **p < 0.010, ***p < 0.001.

## Results

### Clinical and hematological evaluation

**Figure 2.**
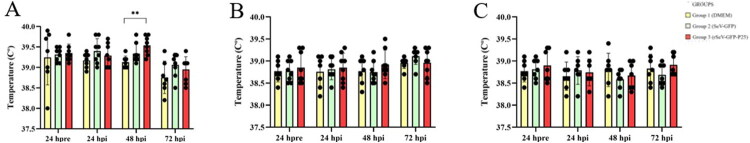
Rectal temperature of the three groups of experimental lambs, taken 24 h before (hpre) and 24, 48 and 72 h after the first (a), second (B) and third (C) immunizations (hpi). Data shown are the mean and standard deviation. Statistically significant differences between groups (**p = 0.004). No statistically significant differences within groups are represented.

Clinical parameters were within reference ranges in all cases (Pugh and Baird 2011). No statistically significant differences were observed between groups, except for temperature (Figure 2). Group 3 showed a significantly higher temperature than group 1, 48 h after the first immunization (*p* = 0.004) (Figure 2A). Group 3 showed a higher temperature 48 h (*p* = 0.049) and a lower temperature 72 h (*p* = 0.007) after the first immunization compared to 24 h before the first immunization (Figure 2A). Group 2 presented a higher temperature pre-immunization than 72 h after the first immunization (*p* = 0.044), and conversely, higher temperature 72 h after the second immunization than 24 h before the second immunization (*p* = 0.018) (Figure 2B). This group also showed lower temperature 48 h after the third immunization than 24 h before the third immunization (*p* = 0.009) (Figure 2C).

Hematological parameters were within the reference intervals (Pugh and Baird 2011) in all lambs at all time-points analyzed (Figure 1). No statistically significant differences between groups were found.

### SeV-GFP neutralizing antibodies

**Figure 3.**
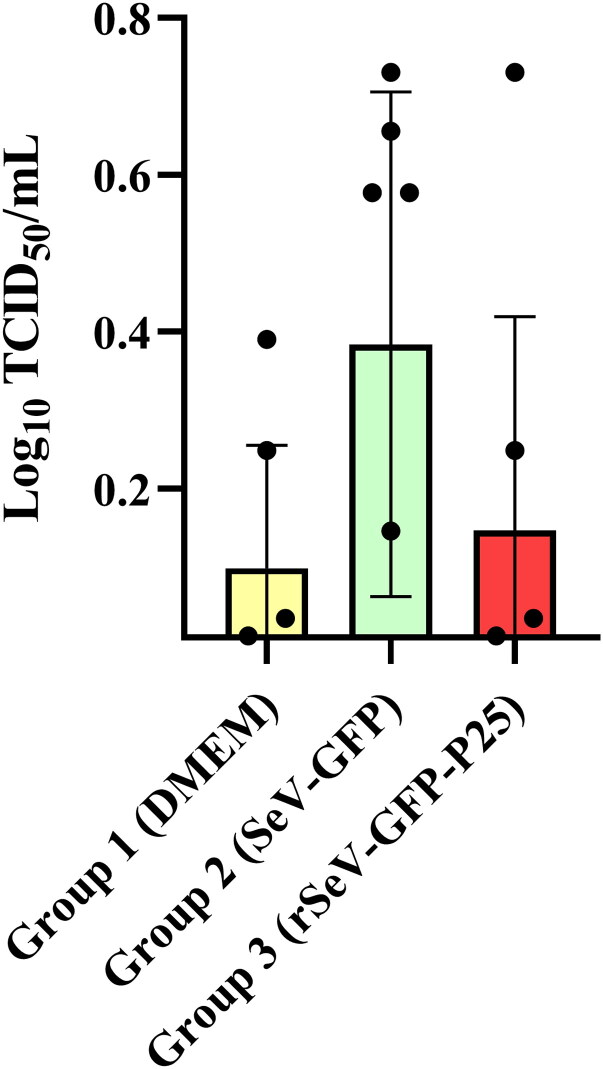
Neutralizing SeV-GFP-specific antibody titers in sera from three groups of experimental lambs, measured 2 weeks after the third immunization (16 wpi). Data shown are the mean and standard deviation.

Group 2 showed four times higher neutralizing SeV-GFP-specific antibody titer than groups 1 and 3, but no statistically significant differences were detected (Figure 3).

### SRLV antibodies

**Figure 4.**
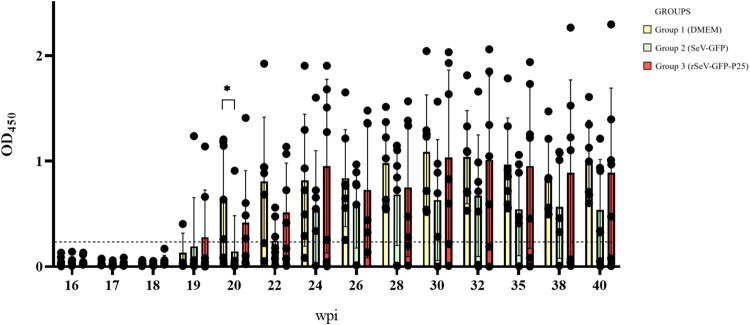
Optical density ratio average values (OD_450_) in anti-SRLV ELISA after challenge (16 weeks post-1^st^ immunization) in lambs of the three experimental groups. Data shown are the mean and standard deviation. Broken line represents the cut-off (0.25). Statistically significant differences between groups (*p = 0.027).

Group 3 showed significantly higher OD_450_ after each immunization: at 5 wpi (*p* = 0.042), 2 weeks after the second immunization (7 wpi) (*p* = 0.046) and third immunization (16 wpi) (*p* = 0.018), when compared to pre-immunization levels (data not shown). At 18, 20 and 22 wpi, groups 3, 1 and 2, respectively, demonstrated a significant OD_450_ increase relative to pre-immunization levels (*p* < 0.05), despite still being considered as seronegative. These significant OD_450_ differences were maintained in the following weeks (*p* < 0.05). The OD_450_ mean exceeded the cut-off at 6 weeks post-challenge (22 wpi) for group 1 and at 8 weeks post-challenge (24 wpi) for group 3 (Figure 4). Four weeks after the challenge (20 wpi), group 1 exhibited significantly higher OD_450_ than group 2 (*p* = 0.029).

First seropositive animals appeared three weeks after SRLV challenge (19 wpi) (Table 1). Although group 1 showed a higher number of seropositive animals after 20 wpi compared with the rest of the groups, no statistically significant differences were observed between groups.

**Table 1.** Number of SRLV seropositive lambs three weeks after challenge (19 weeks post-1^st^ immunization).

GROUPS		Weeks post-1st immunization										
		19	20	22	24	26	28	30	32	35	38	40
1 (DMEM)	*n*	2	5	6	6	7	7	7	7	7	7	7
	%	28.6	71.4	85.7	85.7	100	100	100	100	100	100	100
	ASR	0.4	1.5	1	0.7	1.6	1.6	1.6	1.6	1.6	1.6	1.6
2 (SeV-GFP)	*n*	1	1	4	5	5	5	5	5	5	5	5
	%	14.3	14.3	57.1	71.4	71.4	71.4	71.4	71.4	71.4	71.4	71.4
	ASR	−0.7	−2.2	−1	−0.4	−0.8	−0.8	−0.8	−0.8	−0.8	−0.8	−0.8
3 (rSeV-GFP-P25)	*n*	2	4	5	5	5	5	5	5	5	5	5
	%	28.6	57.1	71.4	71.4	71.4	71.4	71.4	71.4	71.4	71.4	71.4
	ASR	0.4	0.6	0	−0.4	−0.8	−0.8	−0.8	−0.8	−0.8	−0.8	−0.8

ASR: adjusted standardized residuals.

**Figure 5.**
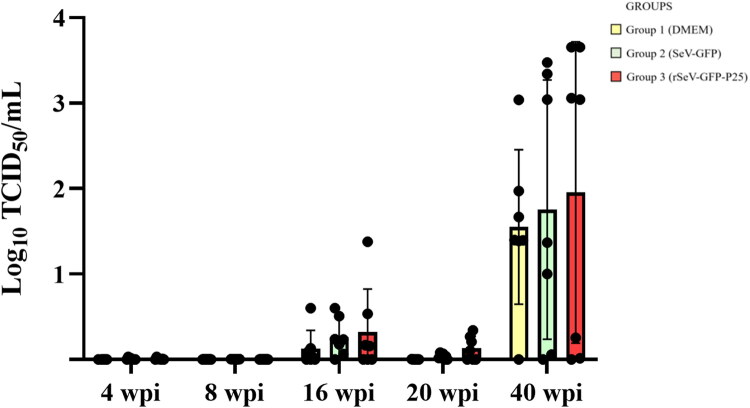
Neutralizing SRLV EV1-strain specific antibody titer in sera from three groups of experimental lambs, measured 4 weeks post-first immunization (wpi), 3 weeks after the second immunization (8 wpi), 2 weeks after the third immunization (16 wpi) and 4 and 24 weeks after the challenge (20 and 40 wpi, respectively). Data shown are the mean and standard deviation. No statistically significant differences within groups are represented.

Group 3 presented higher titer of SRLV neutralizing antibodies compared to other groups in all wpi (Figure 5), but no statistically significant differences were observed between groups. Group 2 and especially group 3 showed higher titers of neutralizing antibodies 2 weeks after the third immunization (16 wpi) (*p* = 0.028) and 24 weeks after the challenge (40 wpi) (*p* = 0.018) compared to 4 wpi. Group 1 presented higher titer of SRLV neutralizing antibodies 24 weeks after the challenge (40 wpi) compared to 4 wpi (*p* = 0.028).

### SRLV DNA quantification in PBMCs

**Figure 6.**
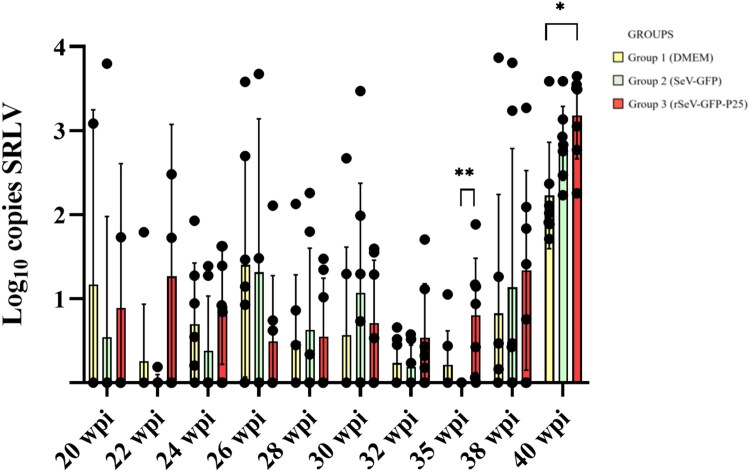
SRLV strain EV1 DNA quantification in peripheral blood mononuclear cells from three groups of experimental lambs starting at 4 weeks after the experimental challenge (20 weeks post-first immunization (wpi)). Data shown are the mean and standard deviation. Statistically significant differences between groups (*p = 0.024, **p = 0.005).

The first detection of SRLV DNA in PBMCs was at 4 weeks after challenge (20 wpi) in all groups (Figure 6). Group 3 showed significantly higher number of SRLV copies than group 2 at 19 weeks after challenge (35 wpi) (*p* = 0.005) and group 1 at 24 weeks after challenge (40 wpi) (*p* = 0.024) (Figure 6).

Group 3 showed a significantly higher number of animals with PBMCs positive to SRLV qPCR, compared to group 2 at 19 weeks after the challenge (35 wpi) (*p* = 0.012) (Table 2). Group 2 had a lower number of positive animals than the other two groups at 4 (20 wpi), 8 (24 wpi), 12 (28 wpi), 16 (32 wpi) and 19 (35 wpi) weeks after the challenge but no statistically significant differences were observed.

**Table 2.** Number of animals with SRLV strain EV1 DNA detected in peripheral blood mononuclear cells 4 weeks after the challenge (20 weeks post-1^st^ immunization).

GROUPS		Weeks post-1^st^ immunization									
		20	22	24	26	28	30	32	35*	38	40
1 (DMEM)	*n*	2	1	5	5	4	2	4	2	5	7
	%	28.6	14.3	71.4	71.4	57.1	28.6	57.1	28.6	71.4	100
	ASR	0.4	−1	0.6	1.2	0.3	−1.2	0	−0.9	0	
2 (SeV-GFP)	*n*	1	2	3	3	3	4	3	1	5	7
	%	14.3	28.6	42.9	42.9	42.9	57.1	42.9	14.3	71.4	100
	ASR	−0.7	0	−1.3	−0.6	−0.6	−0.6	−0.9	−1.9^a^	0	
3 (rSeV-GFP-P25)	*n*	2	3	5	3	4	4	5	6	5	7
	%	28.6	42.9	71.4	42.9	57.1	57.1	71.4	85.7	71.4	100
	ASR	0.4	1	0.6	−0.6	0.3	0.6	0.9	2.8^b^	0	

Statistically significant differences between groups (*p = 0.012).

ASR: adjusted standardized residuals.

^a^ ASR lower than 1.96.

^b^ ASR higher than −1.96.

### SRLV DNA quantification in tissues

**Figure 7.**
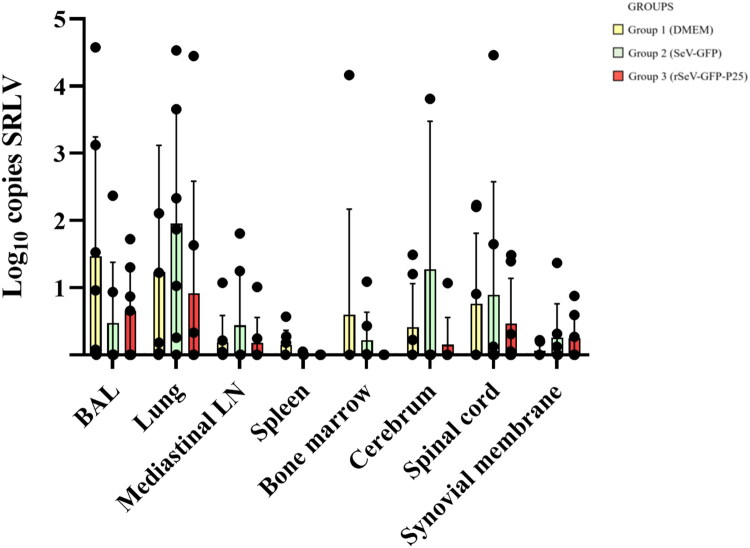
SRLV strain EV1 DNA quantification in tissues from three groups of experimental lambs 25 weeks after challenge (41 wpi). BAL: bronchoalveolar lavage. Data shown are the mean and standard deviation.

Group 3 presented lower copies of SRLV DNA than the other two groups in all tissues, except for the synovial membrane and BAL, where similar proviral loads were found (Figure 7). Group 2 showed the highest number of SRLV copies in lungs, mediastinal lymph node, cerebrum and spinal cord, but no statistically significant differences were observed between groups.

Group 1 presented a significantly higher number of animals with SRLV DNA detection in the spleen compared to groups 2 and 3 (*p* = 0.046) (Table 3). No SRLV DNA was detected in the spleen and bone marrow of animals from group 3. Group 3 showed a lower number of animals than group 1 with SRLV DNA detection in all tissues, except for the synovial membrane and spinal cord.

**Table 3.** Number of animals with SRLV strain EV1 DNA detection in target tissues 25 weeks after challenge (41 weeks post-1^st^ immunization).

GROUPS		BAL	Lung	Mediastinal lymph node	Spleen*	Bone marrow	Cerebrum	Spinal cord	Synovial membrane
1 (DMEM)	*n*	5	5	3	3	2	3	3	2
	%	71.4	71.4	42.9	42.9	28.6	42.9	42.9	28.6
	ASR	1.2	0.3	0.3	**2** ^a^	0.4	1	−0.9	−0.6
2 (SeV-GFP)	*n*	2	6	3	1	3	2	5	3
	%	28.6	85.7	42.9	14.3	42.9	28.6	71.4	42.9
	ASR	−1.5	1.3	0.3	−0.4	1.4	0	0.9	0.3
3 (rSeV-GFP-P25)	*n*	4	3	2	0	0	1	4	3
	%	57.1	42.9	28.6	0	0	14.3	57.1	42.9
	ASR	0.3	−1.6	−0.6	−1.6	−1.8	−1	0	0.3

Statistically significant differences between groups (*p = 0.046).

ASR: adjusted standardized residuals.

^a^ ASR was higher than 1.96.

BAL: bronchoalveolar lavage.

### Pathological findings

**Figure 8.**
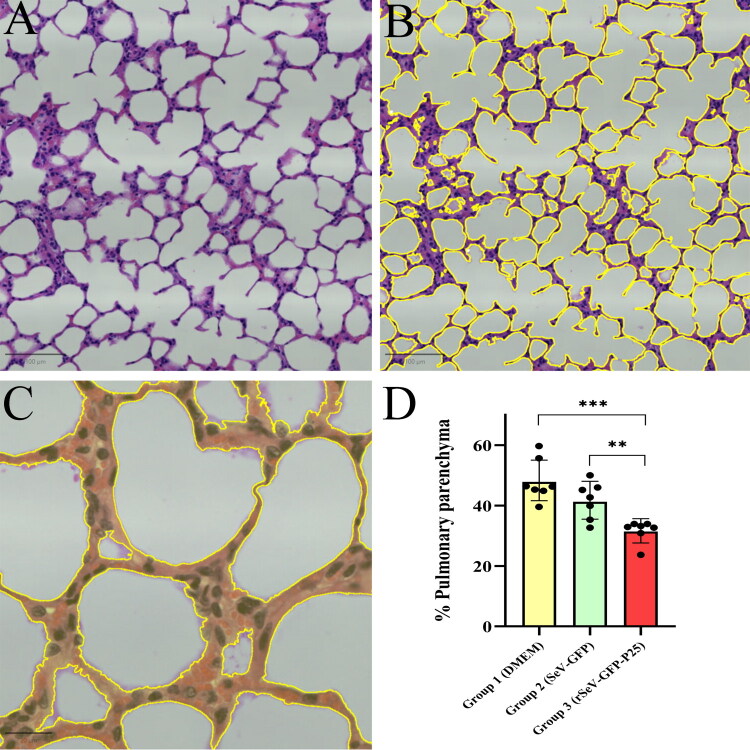
Histomorphometry evaluation of SRLV-associated lung lesions 25 weeks after challenge (41 weeks post-1^st^ immunization). Haematoxylin-eosin. (A) a scanned lung section. (B) Yellow lines represent the limits of the alveolar septum area. (C) Yellow color demarcates the thickness of the alveolar septum area. (D) Percentage of pulmonary parenchyma occupied by alveolar septa. Data shown are the mean and standard deviation. Statistically significant differences between groups (**p = 0.006; ***p < 0.001).

Macroscopic lung lesions associated with SRLV were observed in 3 animals from group 1, and 2 animals from group 2, characterized by an increase in lung volume and consistency. However, no statistically significant differences were detected between the groups. Microscopic semi-quantitative analysis indicated that SRLV-associated lung lesions were mild in 100% (*n* = 7/7) of the animals in group 1, 85.7% (*n* = 6/7) in group 2 and 42.8% (*n* = 3/7) in group 3, with no statistically significant differences in lesion severity between groups. Histomorphometry evaluation revealed a significant increase in the thickness of alveolar septa in group 1 (*p* < 0.001) and group 2 (*p* = 0.006) compared to group 3 (Figure 8). No SRLV-associated microscopic lesions were observed in other tissues, and no tissue reaction was detected in SeV target tissues.

## Discussion

This is the first study to evaluate immune protection against SRLV induced by a recombinant SeV in sheep. Immunization of lambs was performed thrice using recombinant SeV, previously generated (Gómez et al. 2025a). Subsequently, animals were challenged with the homologous SRLV strain EV1 (Saman et al. 1999). Lambs immunized with rSeV-GFP-P25 presented early mild protection against SRLV disease development.

No significant alterations were observed in clinical and hematological parameters, except for the temperature. No fever peaks (temperature over 40 °C) were detected after immunizations. Only group 3, immunized with rSeV-GFP-P25, presented a significant rise in temperature when compared with group 1, 48 h after the first immunization. Moreover, apart from 72 h after the second immunization and 48 h after the third immunization, group 3 always showed a rise in temperature in comparison with group 1, although none of these risings were statistically significant. As described in previous studies, these results confirm that intranasal immunization with SeV-based recombinant vectors induces an immediate mild temperature rise (Kohl et al. 2004; Schulze et al. 2016). Further studies are needed to evaluate rectal temperature measurements 1–2 days post-immunization as a possible indicator of vaccination efficacy (Ahn et al. 2019; Mattiuzzi and Lippi 2022).

Considering SeV-specific neutralizing antibody titers in the three experimental groups, this study suggests a low humoral response against SeV-GFP and rSeV-GFP-P25 in sheep. Group 2 presented four-fold higher SeV-neutralizing antibody titers than groups 1 and 3, but there were no significant differences between them. SeV-specific neutralizing antibody titers observed in the three groups were considered low and probably insufficient to restrict vaccine efficacy, based on results of prior studies (Moriya et al. 2011; Kurihara et al. 2012). As previously described, repeated immunizations with SeV-based vectors may induce the generation of neutralizing antibodies directed to the vector, causing an obstacle for vaccine efficacy, but SeV-based vector inoculation is highly immunogenic despite the presence of previous anti-SeV neutralizing antibodies (Moriya et al. 2008; Yu et al. 2010; Hara et al. 2011; Moriya et al. 2011; Kurihara et al. 2012). Therefore, these results ensure that no significant level of neutralizing antibodies against the SeV vector is induced after repeated intranasal immunizations with rSeV-GFP-P25. In future studies, it would be advisable to develop a quantitative ELISA to determine total antibody titers against SeV-GFP and correlate titers with their neutralizing capacity.

SRLV ELISA results indicate that animals immunized with rSeV-GFP-P25 present a negligible increase of P25-specific antibodies. OD450 increased significantly after every immunization, especially after the third immunization, although with an insufficient antibody content to exceed the ELISA cut-off. Indeed, the first seropositive animals were detected three weeks after the challenge in all groups, suggesting that P25 insert did not induce a robust humoral response. Interestingly, all animals in group 1 were seropositive from 10 weeks after the challenge, whereas only 5 seroconverted in groups 2 and 3. However, the absence of SRLV-specific antibodies in these animals was not correlated with a higher viral load in PBMCs and tissues or with reduced protection. A moderate increase in levels of neutralizing antibodies against homologous SRLV was found 2 weeks after the third immunization, especially in group 3; however, with no statistical significance, probably because of the small number of animals per group and the intrinsic individual variation that is especially high in small ruminants (Schwartz et al. 2019). Remarkably, no neutralizing antibodies were detected in previous studies using other vector-based immunization strategies (González et al. 2005; Niesalla et al. 2009). Group 2 also presented a mild increase of neutralizing antibodies after the third immunization, suggesting a mild cross-reaction. Similar results have been previously obtained using SeV vectors (Gorman and Clark 1990; Lyn et al. 1991; Hara et al. 2011; Ishii and Matano 2015). After the challenge, group 3 continued presenting higher EV1 neutralizing antibodies than groups 1 and 2, although no significant differences were observed. The role of neutralizing antibodies in SRLV infection and immunization is not clear. Experimental infection with SRLV genotype A strains induce neutralizing antibodies but some authors associate them with a more severe pathology, rather than a protective role (Stoskopf and Narayan 1986; Pépin et al. 1998; González et al. 2005; Niesalla et al. 2009; Michiels et al. 2021). Interestingly, specific and neutralizing responses followed different dynamics. Delayed SRLV-specific antibody seroconversion observed in Group 3 was not correlated with neutralization capacity. Conversely, Group 1 showed an earlier seroconversion but delayed neutralization activity.

SRLV DNA copies in PBMCs from group 3 were higher than the other two groups, especially 19 and 24 weeks after challenge, when compared with groups 2 and 1, respectively. Since circulating monocytes are virus carriers in the bloodstream, the increased presence of SRLV DNA copies in blood might reflect the activation and circulation of antigen-presenting cells. Increased infected monocytes may augment extravasation to target tissues and higher tissue viral DNA load would be expected (Blacklaws 2012; Larruskain and Jugo 2013; Michiels et al. 2021). Therefore, this result could suggest that immunization with rSeV-GFP-P25 may favor the spread of SRLV in PBMCs, as described before (Niesalla et al. 2009; Reina et al. 2009). From the technical point of view, non-infectious and defective forms of SRLV DNA, such as imperfect 2LTR circles, could have been amplified by PCR (Coffin et al. 1997; Crespo et al. 2016; Sanjosé et al. 2016). Remarkably, group 3 presented lower copies of SRLV DNA than the other two groups in most SRLV target tissues including spleen and bone marrow, where no SRLV DNA was detected. Spleen and bone marrow have been proposed as infection reservoir tissues (Gendelman et al. 1985; Colitti et al. 2019). SRLV DNA load is a key parameter in tissues to predict lesion development, as the higher the viral load the more probability to develop clinical signs associated with the disease (Wang and McManus 2009). SRLV neutralizing antibodies could inhibit SRLV migration to target tissues and partially control SRLV infection or induce the clearance of the virus in lymphoid tissues (Stoskopf and Narayan 1986; Hooper et al. 2009; Euler et al. 2010; Mikell et al. 2011; Stonos et al. 2014; Liu et al. 2020). Likewise, T cell responses based on CTLs activity could reduce the occurrence of infected cells in tissues, as previously described (Reina et al. 2011). Therefore, our results suggest that immunization with rSeV-GFP-P25 can reduce the viral load in reservoir tissues at early stages. Whether the absence of SRLV DNA in certain target tissues is the consequence of an active CTLs activity after the generation of tissue-resident memory T cells warrants further studies.

Animals in our experiment were euthanized approximately 6 months after the intratracheal challenge. It is well known that SRLV induce chronic and progressive lesions, usually appearing years after infection (Minguijón et al. 2015; Pinczowski et al. 2017). In this study SRLV-associated macroscopic lung lesions were observed in 3 animals from group 1 and in 2 animals from group 2. Interestingly, no macroscopic lesions were observed in group 3. Microscopically, most of the animals exhibited mild SRLV-associated pulmonary lesions, including the thickening of the alveolar septa, interpreted as interstitial pneumonia (Pinczowski et al. 2017). The short interval between the challenge and euthanasia resulted in minimal lesion development, and no significant differences in lesion severity were observed between groups when histopathological samples were observed by pathologists. Therefore, histomorphometry analysis was applied to the same samples and group 3 showed a significantly lower thickening of alveolar septa compared to the other two groups. These results clearly suggest that immunization with rSeV-GFP-P25 provides mild protection against the early progression of interstitial pneumonia. Histomorphometry with applied AI are excellent tools to help increase the sensitivity of the histopathological diagnosis and demonstrate subtle lesions like the ones observed in the present study (Bankhead et al. 2017). Strains from genotype A, such as the EV1 strain, are more prone to display lung or mammary lesions, whereas genotype B strains have been more related to arthritis development (Sargan et al. 1991; Ramírez et al. 2013; Olech 2023). Future research will clarify if the mild protection against SRLV disease observed here after homologous challenge could also be achieved against genotype B of SRLV. Remarkably, no SRLV or vaccine associated lesions were observed in other tissues.

In summary, intranasal immunization with rSeV-GFP-P25 in sheep conferred a minimal SRLV-specific antibody response, reduced viral load in reservoir tissues, and induced early and mild protection against homologous SRLV-associated interstitial pneumonia. Furthermore, this study demonstrates that SeV-based vectors appear to be highly safe in sheep. Therefore, these findings encourage further investigation into whether the cellular immune response is responsible for the partial protection observed, as well as the assessment of long-term immunization in future experiments.

## Supplementary Material

Supplementary material.pdf
