# Supplementary material for: Immunogenicity, security and protection against small ruminant lentivirus (SRLV) challenge in sheep, induced by intranasal immunization with a recombinant Sendai virus vector expressing SRLV gag-P25
Source: Vet Q. 2025 Sep 17;45(1):1–16. doi: 10.1080/01652176.2025.2556492 (PMC12444960; doi:10.1080/01652176.2025.2556492)
Supplement: Supplementary material.pdf [file TVEQ_A_2556492_SM8275.pdf]

Supplementary material

Immunogenicity, security and protection against small ruminant lentivirus (SRLV) challenge in sheep, induced by intranasal immunization with a recombinant Sendai virus vector expressing SRLV *gag*-P25

Álex Gómez[1,2], Idoia Glaria[3], Irati Moncayola[3], Irache Echeverría[4], Ana Rodríguez-Largo[5], Ignacio de Blas[1,2], Estela Pérez[1,2], Marta Pérez[2,6], Sergio Villanueva-Saz[1,2], Benhur Lee[7], Alicia de Diego[8], Ricardo de Miguel[9], Lluís Luján[1,2], Ramsés Reina[3]*

[1]Departamento de Patología Animal, Universidad de Zaragoza, Zaragoza, Spain

[2]Instituto Agroalimentario de Aragón-IA2, Universidad de Zaragoza, Zaragoza, Spain

[3]Instituto de Agrobiotecnología (CSIC-Gobierno de Navarra), Mutilva Baja, Navarra, Spain

[4]Departamento de Agronomía, Biotecnología y Alimentación, Universidad Pública de Navarra, Pamplona, Spain

[5]Servei de Diagnòstic de Patologia Veterinària, Departament de Sanitat i Anatomia Animals, Universitat Autònoma de Barcelona, Barcelona

[6]Departamento de Anatomía, Embriología y Genética Animal, Universidad de Zaragoza, Zaragoza, Spain.

[7]Department of Microbiology, Icahn School of Medicine at Mount Sinai, New York, New York, USA

[8]Instituto Aragonés de Ciencias de la Salud (IACS), Centro de Investigación Biomédica de Aragón (CIBA), Zaragoza, Spain

[9]AnaPath Services GmbH, Liestal, Switzerland

Supplementary Table S1. Semiquantitative score for histopathological severity of SRLV-associated lung lesions.

Parameter	Scoring	Description
Interstitial pneumonia	0 to 3	0: Absence<br>1: Mild, multifocal to coalescence lymphocytic infiltrate thickening mildly the alveolar septum<br>2: Moderate, multifocal to coalescence lymphoplasmacytic and macrophagic infiltrate thickening moderately the alveolar wall<br>3: Severe, diffuse lymphoplasmacytic and macrophagic infiltrate thickening severely the alveolar septum
Intralveolar macrophages	0 to 3	0: Absence<br>1: Multifocal, low number of intralveolar macrophages<br>2: Multifocal to coalescence, moderate number of intralveolar macrophages<br>3: Diffuse, high number of intralveolar macrophages
BALT* hyperplasia	0 to 3	0: Absence<br>1: Mild, multifocal lymphoid hyperplasia<br>2: Moderate, multifocal lymphoid hyperplasia<br>3: Severe, multifocal lymphoid hyperplasia, occasionally forming tertiary lymphoid follicles
Perivasculitis	0 to 3	0: Absence<br>1: Mild, multifocal lymphocytic infiltrate<br>2: Moderate, multifocal lymphocytic and macrophagic infiltrate<br>3: Severe, multifocal lymphocytic and macrophagic infiltrate with occasional fibrosis

*BALT: bronchus-associated lymphoid tissue.
